# Transcranial Stimulation of the Orbitofrontal Cortex Affects Decisions about Magnocellular Optimized Stimuli

**DOI:** 10.3389/fnins.2017.00234

**Published:** 2017-04-26

**Authors:** Anna Bognár, Gergő Csete, Margit Németh, Péter Csibri, Tamás Z. Kincses, Gyula Sáry

**Affiliations:** ^1^Department of Physiology, University of SzegedSzeged, Hungary; ^2^Department of Neurology, University of SzegedSzeged, Hungary; ^3^Department of Anaesthesiology and Intensive Therapy, University of SzegedSzeged, Hungary

**Keywords:** tDCS, OFC, categorization, magnocellular pathway, top-down

## Abstract

Visual categorization plays an important role in fast and efficient information processing; still the neuronal basis of fast categorization has not been established yet. There are two main hypotheses known; both agree that primary, global impressions are based on the information acquired through the magnocellular pathway (MC). It is unclear whether this information is available through the MC that provides information (also) for the ventral pathway or through top-down mechanisms by connections between the dorsal pathway and the ventral pathway via the frontal cortex. To clarify this, a categorization task was performed by 48 subjects; they had to make decisions about objects' sizes. We created stimuli specific to the magno- and parvocellular pathway (PC) on the basis of their spatial frequency content. Transcranial direct-current stimulation was used to assess the role of frontal areas, a target of the MC. Stimulation did not bias the accuracy of decisions when stimuli optimized for the PC were used. In the case of stimuli optimized for the MC, anodal stimulation improved the subjects' accuracy in the behavioral test, while cathodal stimulation impaired accuracy. Our results support the hypothesis that fast visual categorization processes rely on top-down mechanisms that promote fast predictions through coarse information carried by MC via the orbitofrontal cortex.

## Introduction

Fast decisions about environmental information require categorization to distinguish between animate and non-animate things, plants and animals, vehicles and buildings, etc. (Fabre-Thorpe, 2011). Categorization serves not only distinction but also generalization when different objects are grouped on the basis of shared features (Keller and Soenfeld, 1950). The visual environment does not always favor perception: fog, poor lighting, absence of colors, low contrast, short flashes of an image allow only decisions made on the basis of coarse, global features or outlines of objects. In addition, sometimes only the periphery of the visual field is stimulated; still, we need to know whether this visual information has any relevance. For a detailed analysis on the other hand, fine details, colors and edges are important.

For fast and efficient categorization relevant information and actual goals should be considered. This process might root in the two major visual processing streams: the magnocellular pathway (MC) and the parvocellular pathway (PC). The majority of axons leaving the retina belong to either the MC or the PC. The MC runs (partly) to the frontal lobe, while the end of the PC stream is in the inferotemporal cortex (IT), a region essential for visual recognition. Instead of a detailed description (but see e.g., Mishkin and Ungerleider, 1982; Goodale and Milner, 1992) of the fundamental differences in the properties of the MC and the PC, here we focus only on those features of the MC which are relevant to our study. The MC pathway is very fast. Differences in conduction speed between the two pathways can be demonstrated as early as the lateral geniculate body (LGB): information arriving via the PC has some 20 ms delay as compared to the MC, and this difference is also present in V1 (Maunsell and Newsome, 1987; Nowak et al., 1995; Schmolesky et al., 1998). After V1 it takes only 6–9 ms to reach V3, the middle temporal area (MT), the middle superior temporal area (MST) or the frontal eye field (FEF) (Schmolesky et al., 1998).

On the basis of latency differences between the PC and the MC, Nowak and his colleagues suggested that visual signals processed in the MC might modulate activity in the PC through feed-forward, lateral or feed-back connections (Nowak and Bullier, 1997). Information carried rapidly by the MC toward the frontal areas may exert a top-down effect. In contrast with the hierarchical views of visual processing, this top-down effect is supposed to be able to modulate lower regions from higher cortical areas which have been activated earlier (Knierim and van Essen, 1992; Zipser et al., 1996). However, due to the fact that the MC is sensitive only to coarse features, the role of the MC in object recognition was not investigated for long. Recently published papers, however, suggest that when time is an issue, the MC carries sufficient data to extract relevant information, which—provided there is enough time—can be completed by colors and details carried by the PC. Several experiments (see below) were carried out in order to investigate rapid categorization by using pathway-specific stimulation.

Research on decisions concerning MC information can benefit from the fact that images projected on the peripheral retina almost exclusively stimulate the rod system. In a study by Thorpe and colleagues (Thorpe et al., 2001), participants had to decide about images and choose between animate/non-animate categories. Their results showed that eccentricity did not have an influence on the accuracy of the decisions and that low spatial frequency (LSF) information originating from the periphery of the retina was sufficient for categorization. It was also shown that rapid categorization is possible in the absence of colors (Delorme et al., 2010). The MC is sensitive to the achromatic differences in luminance; the pathway can be stimulated by stimuli having low (<8%) contrast and LSF (Tootell et al., 1988). Experiments on monkey and human participants using contrast differences (Mace et al., 2005, 2010) were performed and showed that images with sufficiently low contrast are invisible for the PC, so decisions concerning the stimuli *must* be based on information carried by the MC. If the PC were the only pathway involved in visual categorization, low contrast stimuli should cause a dramatic decrease in performance. However, at contrast values of 3% performance did not change significantly in either species, which suggests that it might be done on the basis of coarse information carried by the MC (Bar et al., 2001; Bar, 2003).

Different spatial frequencies carry different aspects of the visual stimuli. High spatial frequencies (HSFs) carry information about edges and patterns, while LSFs contain global information. The latter might be sufficient to make a first, global impression about the general shape of objects. Psychophysical studies show that LSF patterns (Sachs et al., 1971; De Valois et al., 1990) and complex sceneries (Schyns and Oliva, 1994; Mace et al., 2005, 2010) are perceived earlier than high SF. Electrophysiological results show that the first part of the activity of IT cells reflects global information (Sugase et al., 1999; Tamura and Tanaka, 2001) and only the later part of the responses, after some 51 ms, carries information about fine details (Sugase et al., 1999). This means that IT neurons respond first to low LSF and global features and only after that to fine details.

According to the studies mentioned above and based on their EEG findings, Thorpe and Fabre-Thorpe suggested an MC based, fast pathway which uses the same cortical areas as the ventral pathway. Thus, MC information arrives at the IT faster and reaches the prefrontal cortex and the motor cortex earlier than information carried by the PC if a fast decision is needed (Fabre-Thorpe et al., 2001; Thorpe and Fabre-Thorpe, 2003). Reaction times in monkeys performing rapid visual categorization are as short as 180 ms, which leaves time only for a feed-forward processing through the IT to the motor cortex via the prefrontal and premotor cortices (Fabre-Thorpe et al., 1998). It was also suggested that MC information supported PC processing through fast, local feed-back circuits along the ventral visual stream (Fabre-Thorpe, 2011).

Bar and his colleagues, on the other hand, hypothesized a top-down process which, using the rapid processing in the MC through the dorsal pathway could provide the IT with coarse but fast information through the orbitofrontal cortex (OFC). This top-down mechanism can limit the number of possible interpretations, decrease the amount of necessary computation and reduce the time needed. This global information is essential for making fast decisions for survival (Bar, 2003). In these experiments, the two pathways were stimulated selectively and categorization was required (Bar, 2003; Kveraga et al., 2007a,b). According to the findings, the critical structure in top-down processes is the OFC, whose early activation can be attributed to processing visual information in the MC (Bar, 2003; Kveraga et al., 2007b). In addition, a study investigating the functional coupling of cortical areas found phase coupling between V1 and the OFC, and the OFC and the IT (see Lin et al., 2004). Rokszin et al. (2016) investigated how the top-down effects are manifested in scalp ERPs when presenting low or high SF information. They found evidence of top-down, anterior effect for MC optimized images within the first 200 ms of visual processing (shorter N1 latencies and amplitude changes spreading to anterior scalp regions). The connection is provided by the fibers of the uncinate fascicle and the external capsule connecting the OFC with the IT (Cavada and Goldman-Rakic, 1989; Cavada et al., 2000; Fang et al., 2005).

**Figure 1:**
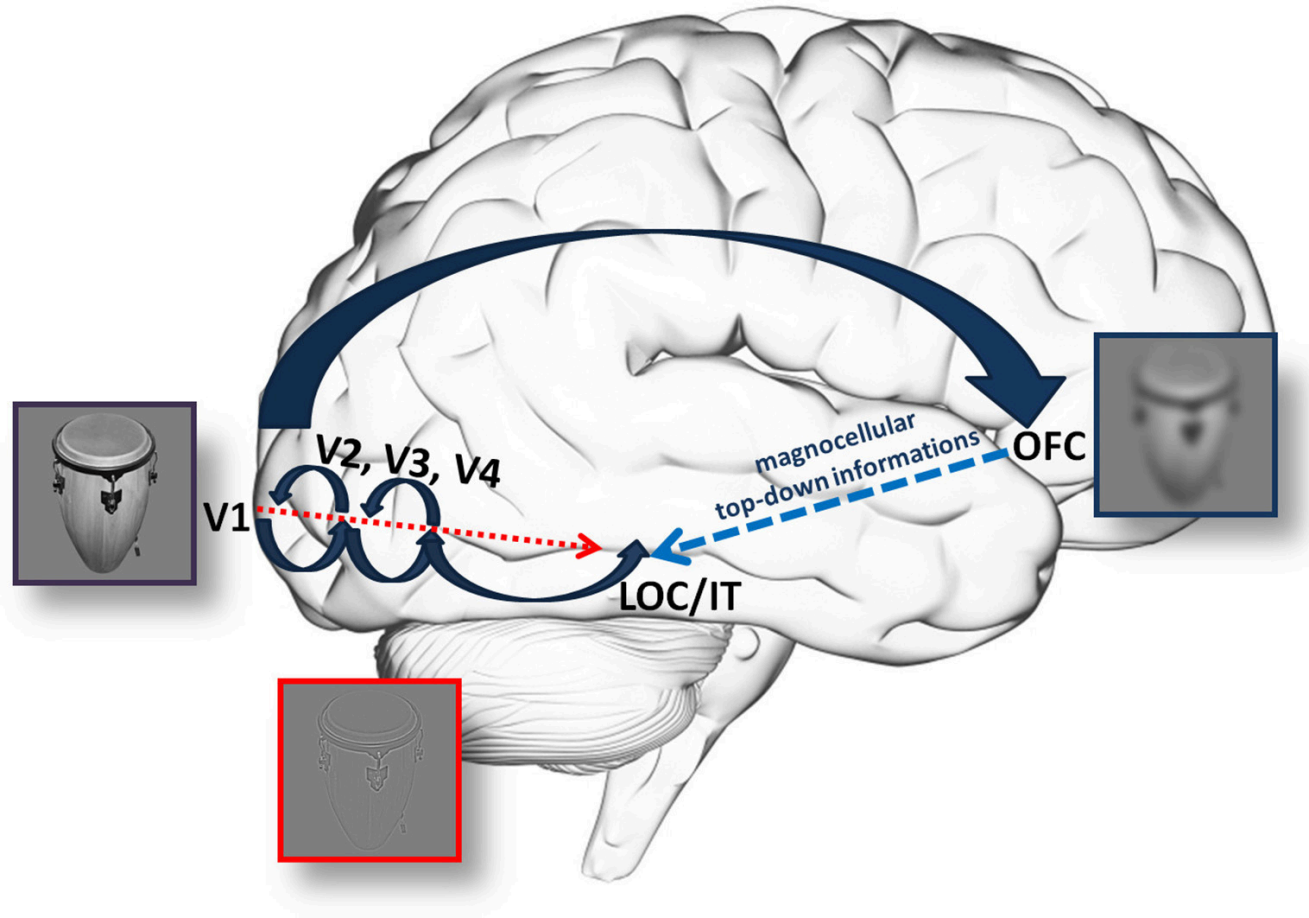
**An illustration of the hypothetical anatomical background for information processing through the (fast) magnocellular and parvocellular pathway**. According to Fabre-Thorpe (2011), MC information supports PC processing through fast, local feed-back circuits. On the other hand, Kveraga and his colleagues hypothesized a top-down process, which, using the rapid processing in the MC, could provide the IT through the OFC with fast but coarse information. This can feed-back to the ventral stream to limit the number of possible interpretations, decrease the amount of necessary computation and the time needed. Please note, that arrows merely indicate a supposed, general flow of information and not necesseraly anatomical stages. This is especially true for large arrow indicating the dorsal pathway, where the route of information is not yet clear.

It is important to note that although the MC is regarded as the main input for the dorsal or “Where?” pathway processing motion and serving spatial attention, nearly 50% of the MC fibers feed information into the ventral stream (Ferrera et al., 1992; Nealey and Maunsell, 1994). There is plenty of evidence supporting the role of the MC pathway in fast categorization; however, it is unclear whether this information after leaving V1 reaches the IT via the dorsal (a top-down process through the OFC) or the ventral pathway (local feed-forward or feed-back circuits preceding PC information) (Figure 1).

The goal of our study was to determine which of the above scenarios is more likely: does MC information responsible for fast visual decisions pass through the OFC or does it run together with the ventral pathway? One possible approach of the problem might be to interfere with the dorsal or ventral pathway to see whether the processing of those stimuli which are characteristic to the given pathway is affected or not. A logical choice is a non-invasive and reproducible electrical stimulation of the pathway(s).

Electrical stimulation manipulates the activity of cortical networks transitionally and reversibly in a non-invasive and painless way. The method consists of a weak transcranial current (tDCS) flowing through the brain using two large surface electrodes (Nitsche and Paulus, 2000; Manuel et al., 2014), which can influence cortical functions. In the past few years several studies investigated visual processing in humans using non-invasive electrical stimulation to directly modulate visual cortices in human subjects (Antal et al., 2001). The anodal stimulation over V1 increases the sensitivity of phosphenes (Antal et al., 2003a), contrast sensitivity, enhances the amplitude of N70 while the opposite effects were found using cathodal stimulation (Antal et al., 2003b,c, 2004a; Kraft et al., 2010). Futhermore, tDCS modulates human color discrimination in a pathway-specific manner (Costa et al., 2012). The anodal stimulation over MT improves learning of visually guided tracking movements (Antal et al., 2004c). After learning the anodal stimulation has no effect, but cathodal stimulation can increase the signal-to-noise ratio and improve the performance in the learned task (Antal et al., 2004b). The tDCS over the posterior parietal cortex modulates visuospatial processing (Sparing et al., 2009), bilateral stimulation over the anterior temporal lobe (right anodal, left cathodal) improves visual memory (Chi et al., 2010), cathodal stimulation of the temporo-parietal cortex reduces the magnitude of facial adaptation (Varga et al., 2007). Also, anodal stimulation improves implicit learning when the left prefrontal cortex is stimulated (Kincses et al., 2004) and enhances the recognition of facial expression when right OFC is stimulated (Willis et al., 2015). For a review see Antal et al. (2011) and Costa et al. (2015).

Effects of tDCS might be explained by the modulation of the resting membrane potentials of the stimulated area. Single cell recording studies have shown that cathodal stimulation can decrease firing activity, while the anodal stimulation have the opposite effect (Bindman et al., 1964; Purpura and McMurtry, 1965). In humans the tDCS has similar polarity dependent effects (Nitsche and Paulus, 2000, 2001). It seems that tDCS effects appear to be site specific but not site limited; the latter effects might be based on plasticity mechanisms.

Since tDCS seems to be a powerful technique for investigation visual processing, we applied cathodal or anodal tDCS and sham stimulation as a control in a decision making test, over the OFC (Nitsche et al., 2008; Dayan et al., 2013; Manuel et al., 2014; Willis et al., 2015). Our subjects were required to make a judgment on the real size of objects seen on the screen, i.e., whether they fit in a shoebox or not? There were two sessions; between the two sessions tDCS stimulation was applied.

There are two possible scenarios concerning the outcome. If stimulation of the OFC does not have an effect on decisions concerning *both* MC *and* PC optimized stimuli, or if the effects are similar using *both* stimuli that would support the idea that fast MC information is processed through the ventral pathway avoiding the OFC. Thus, only *decision mechanisms* were affected, but not the *route of information flow*. If, on the other hand, decisions about MC stimuli were affected selectively, it would support the hypothesis that MC information reaches the OFC, passes through it and is available for top-down modulation (Bar et al., 2006).

## Materials and methods

### Stimuli

**Figure 2:**
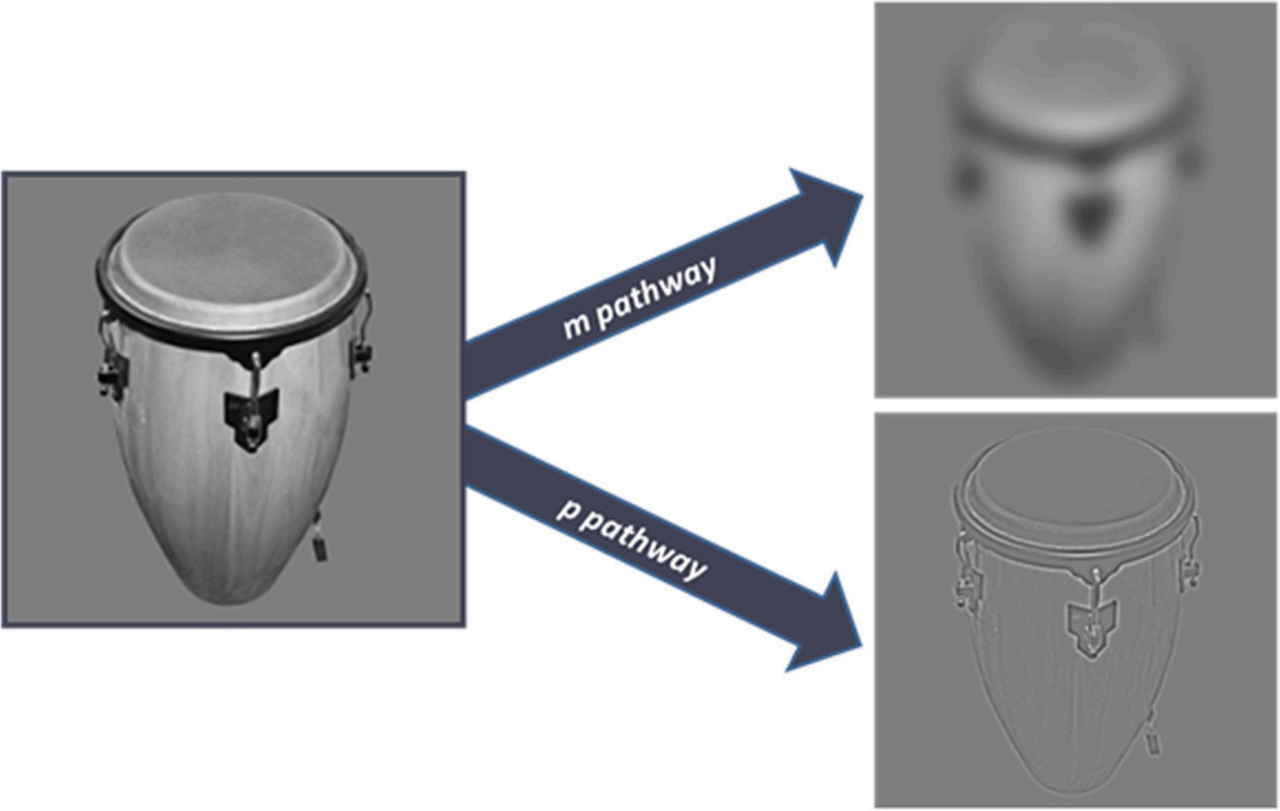
**The image on the left is the original unfiltered image of an object received by the retina**. The right side of the figure shows the two kinds of stimuli used in the experiment. The upper image is filtered for the selective stimulation of the magnocellular pathway. The bottom image is optimized for the ventral stream, in accordance with the sensitivity of the parvocellular pathway.

The stimulus set contained 200 achromatic images of everyday objects, like a truck, ashtray, pen, piano, etc. One part of the images was collected from the Bank of Standardized Stimuli (Brodeur et al., 2010) others were selected and collected by one of the authors (A.B.). Stimuli were modified using Matlab and GIMP 2.8 programs. Stimuli were cut out from the original pictures, were standardized in the sense that all had the same size in their largest dimension (4,5° viewed from 57 cm) placed on the same background, transformed to grayscale images. Shine Toolbox was used to equalize the contrast and luminance values before filtering (Willenbockel et al., 2010). Images had resolutions of 72 pixels per inch and size of 500^*^500 pixel. The visual stimuli were modified to selectively stimulate the MC or the PC; they were filtered by Gaussian filter (12 pixel kerner, as lowpass filter) and highpass filter (0.5 radius) to attenuate the high and spatial frequencies, respectively. The MC optimized stimuli contained LSF (<0.9 cycles per degree), while the PC stimuli consisted of HSF (>4.7 cycles per degree, Figure 2). This method is similar to the one used by Bar et al. (2006). All stimuli had a mean luminance between 8 and 9 cd/m^2^. No luminance matching was used after filtering. The images of the objects could be divided into two groups according to their real life size. One half of the objects were larger, while the others were smaller than an average shoe box. All stimuli were presented on a uniform gray background (8.9 cd/m^2^). For stimulus presentation a 23-inch LCD (Tobii Pro TX300) monitor was used having screen resolution of 1,920 × 1,080 and vertical refresh rate of 60 Hz.

### Subjects

Forty-eight healthy subjects (university students, 19 females; mean age: 22.7 years) participated in the study. They were divided in three equal groups for cathodal, anodal and sham stimulation. Each subject had to perform the task before and after the stimulation (see below). All had normal or corrected-to-normal vision, including normal color vision and none of them suffered from any neurological or psychiatric disorders. None of them had a history of excessive drug/alcohol/caffeine consumption. A questionnaire was provided regarding previous diseases, handedness (Oldfield, 1971), sleep time, medication, mental and physical status. All study participants gave written informed consent in accordance with the Declaration of Helsinki; the study was approved by the ethical committee of the University of Szeged (Ref. no.: 165/2014).

### Behavioral test

The subjects were seated in a sound-attenuated, dimly lit room, and viewed the computer screen from 57 cm. For stimulus presentation a custom made MATLAB code (MathWorks, Natick) and the Psychtoolbox Version 3 (Brainard, 1997) was used.

**Figure 3:**
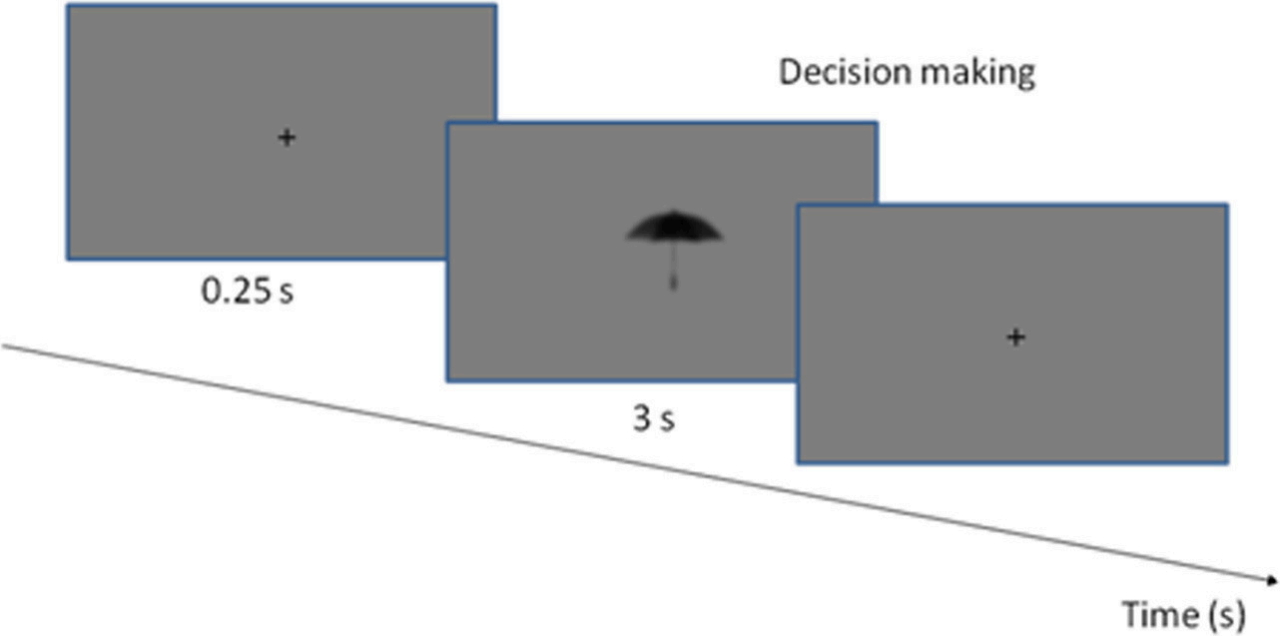
**The experimental procedure**. The stimuli and the fixation point were presented in a gray background. Each trial started with the presentation of a fixation cross, which was visible for 0.25 s. The stimulus was presented until the decision was made, or up to 3 s.

At the beginning of the experimental procedure all subjects received instructions on the computer screen to make sure that everyone was given identical instructions on how to solve the task. There were two sessions during the test, thus each subject was tested twice. In the first session, before the tDCS, half of the stimulus set (100 images) was presented, which contained an equal number of small, large, MC and PC optimized object images in a pseudorandom order. The second session started just after tDCS (or the sham stimulation) and the rest of the stimuli (other 100 images) were presented again in a pseudorandom order. During the psychophysical sessions, the participants were required to make decisions about the object size and to answer the question whether the object displayed on the screen was larger or smaller than a shoebox (Kveraga et al., 2007a). The left arrow key on the computer keyboard was associated with smaller, the right arrow key with larger objects. Size decisions were tested in a preliminary psychophysical experiment. The trials started with a centrally presented fixation-cross (250 ms) appearing before the stimulus in the center of the screen followed by the test stimulus. The trials were machine paced: if no response key was pressed for 3 s, the next image was presented. There was no feedback on the correctness of the responses (Figure 3).

### Stimulation protocol

To modulate prefrontal cortical activity, transcranial direct current stimulation was applied (Kincses et al., 2004; Nitsche et al., 2008; Manuel et al., 2014). Two rubber electrodes (surface: 5 × 7 cm) were used with a neuroConn DC-stimulator (neuroConn GmbH). The electrodes were arranged according to the study of Manuel et al. (2014). They reported a significant modulation of the OFC function (reality filtering) upon direct current stimulation. In their study, the electrical fields induced by tDCS were modeled to predict whether significant current reached the OFC. The model reached a significant current flow in the OFC when the electrodes were placed over the glabella and the vertex (Fpz and Cz of the 10–20 EEG system, respectively) and the electrical field values were calculated for 1 mA of inward current. In our study, the electrodes were placed on the midline; the center of the relevant active tDCS electrode was over the putative OFC cortex (Fpz), while the reference electrode was over the vertex (identified by the standard 10–20 system). Modulation was applied for 20 min with 1 mA current intensity using 10 s fade in and fade out phase in cathodal and anodal stimulation protocol, respectively. Sham stimulation consisted of placing the electrodes on the skull, but no tDCS was applied with the exception of the 10 s fade in and 10 s fade out phases. This stimulation does not have any effect on cortical excitability, but causes the same itching sensation under the electrodes. The total duration of the sham phase was also 20 min. The study was a single-blind experiment: the experimenter was fully informed, but participants were not informed about the type of stimulation they received.

### Statistics

**Figure 4:**
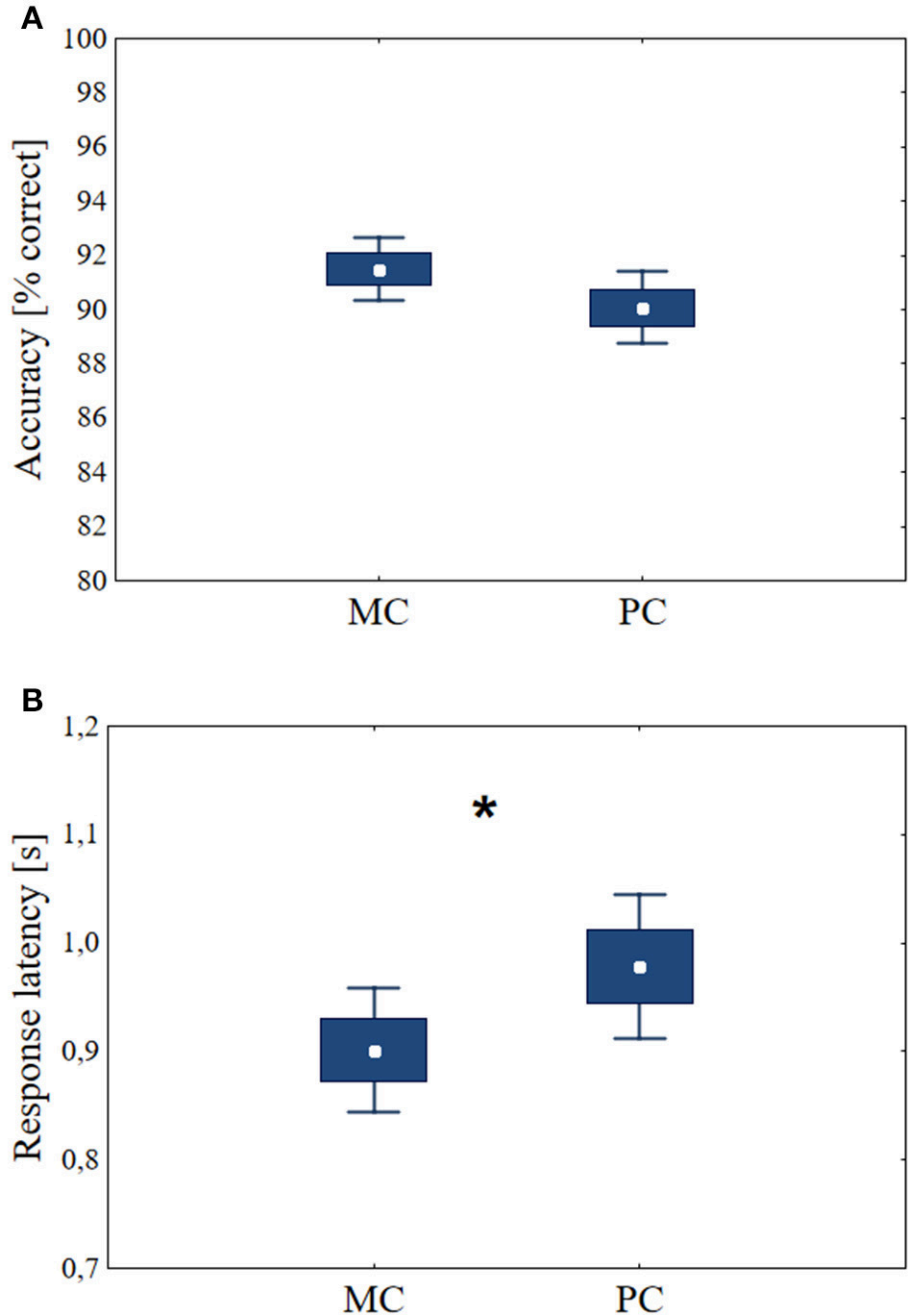
**The accuracies and response latencies during the decision task before tDCS**. Central data points: means, boxes: mean ± SE, bars: mean ± 1.96 SE. **(A)** There was no significant difference between decisions about stimuli optimized for the MC and the PC. **(B)** For MC stimuli, the response latencies are shorter than for PC stimuli (*n* = 48, *p* < 0.01). Asterisk indicates significant differences (*p* < 0.05).

**Figure 5:**
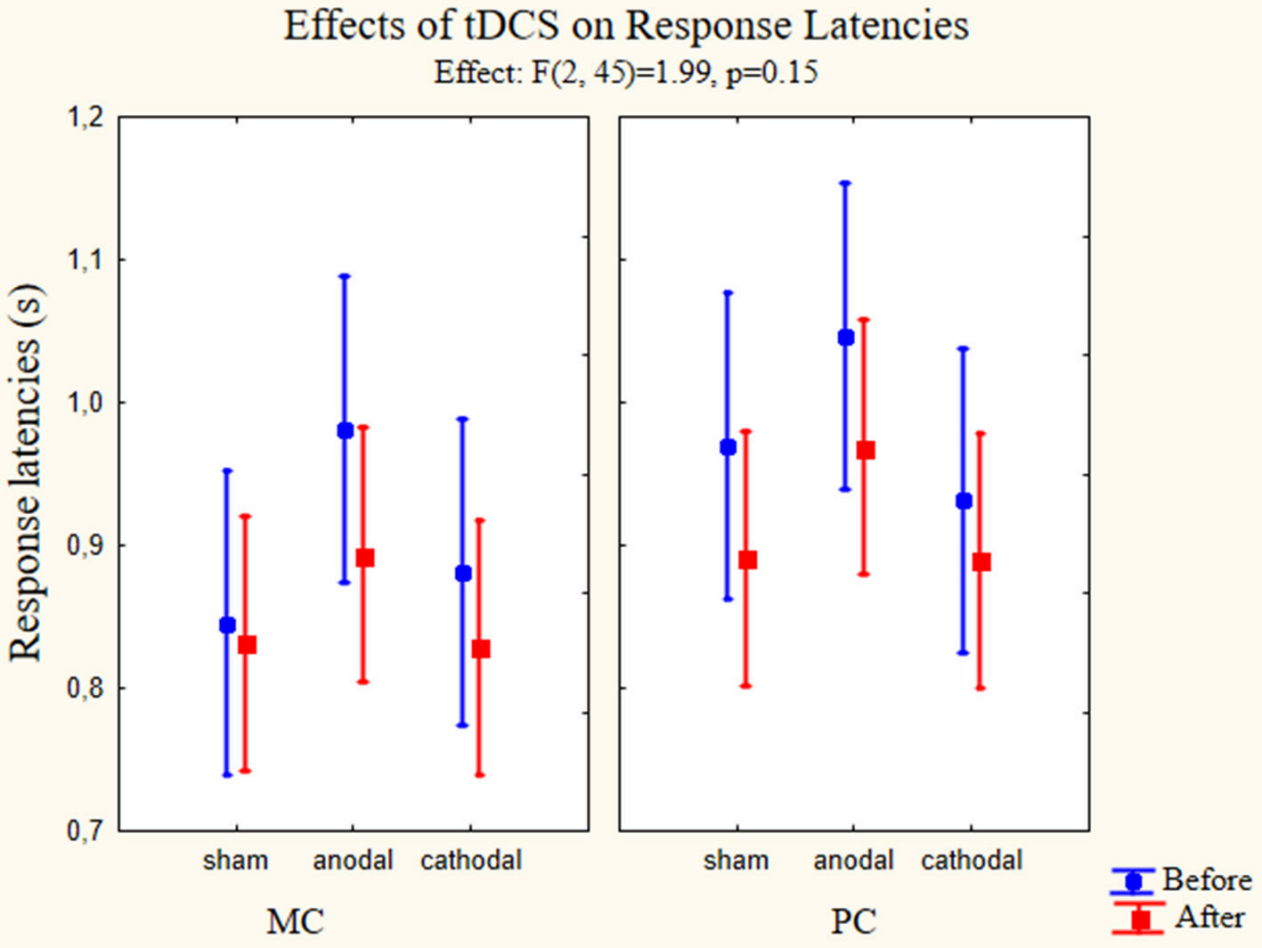
**Effects of tDCS on response latencies**. Repeated measures three-way ANOVA results of the response latencies in the psychophysical tests (*n* = 48). On the left panel the response latencies for MC optimized stimuli are presented. On the right panel we presented the values measured using PC optimized stimuli. Full circles show the measured latencies before stimulation, full squares show the response latencies after stimulation. Data points denote means, vertical bars show 0.95 confidence intervals. None of the stimulation types affected the response latencies.

**Figure 6:**
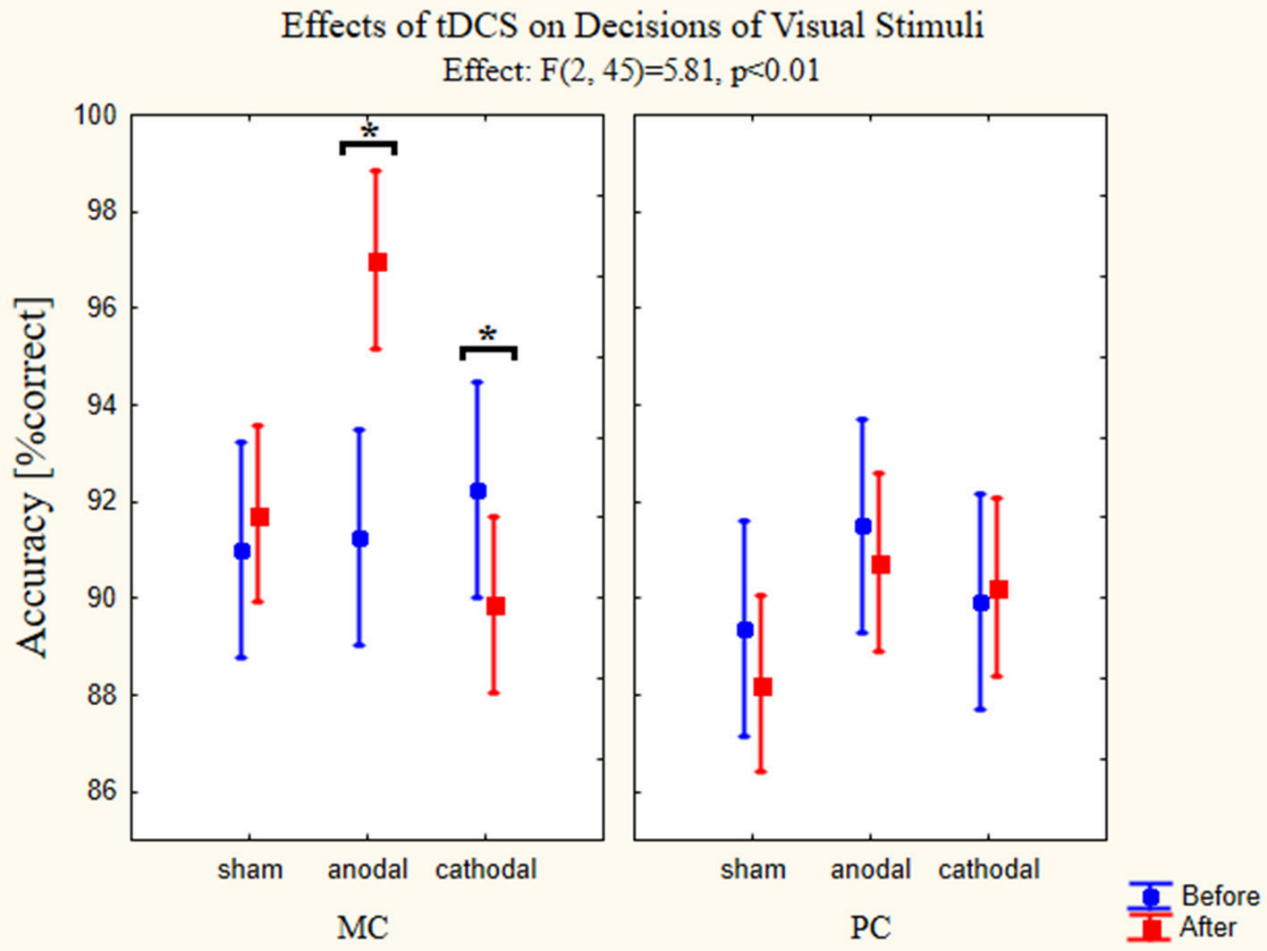
**Effects of tDCS on decisions of visual stimuli**. Repeated measures three-way ANOVA results of the accuracies in the psychophysical tests are presented on the figures (*n* = 48). (full circles: before stimulation, full squares: after stimulation). The left panel presents the accuracy changes using MC optimized stimuli. Anodal tDCS resulted in a better accuracy for these images, while the cathodal stimulation impaired the performance. Sham stimulation did not have any effect on the accuracy. On the right panel accuracies in the psychophysical tests for PC optimized stimuli are shown. None of the stimulation types affected the performance. Data points denote means, vertical bars show 0.95 confidence intervals. Asterisk indicates significant differences (*p* < 0.05).

To see the differences in processing time for the MC and PC optimized stimuli, SPSS Inc. software was used to compare response latencies and accuracies before stimulation (since the conditions were the same for each participant in this period); a paired *t*-test was applied, differences were considered as significant if the type I. error was <0.05. To evaluate the effects of transcranial stimulation we used repeated measures three-way ANOVA with between group factors being type of stimulation and within group factors being time of behavioral test, and pathway (MC, PC). We compared the response accuracy and the reaction times before and after the stimulation. Group averages and standard errors are shown in Table 1, comparisons in Figures 4–**6**.

**Table 1:** **Means of accuracies and reaction times with their confidence intervals in each condition**.

**Stimulation type**			**Means**	**Confidence intervals**
Sham *n* = 16	I.	PC optimized reaction time	0.97	0.86–1.08
		PC optimized performance	89.25	87.05–91.45
		MC optimized reaction time	0.85	0.74–0.95
		MC optimized performance	91.00	88.80–93.19
	II.	PC optimized reaction time	0.89	0.80–0.98
		PC optimized performance	87.73	85.94–89.53
		MC optimized reaction time	0.83	0.74–0.92
		MC optimized performance	91.75	89.95–93.54
Cathodal *n* = 16	I.	PC optimized reaction time	0.93	0.82–1.04
		PC optimized performance	89.81	87.61–92.01
		MC optimized reaction time	0.88	0.77–0.99
		MC optimized performance	92.25	90.05–94.45
	II.	PC optimized reaction time	0.89	0.80–0.98
		PC optimized performance	90.24	88.44–92.03
		MC optimized reaction time	0.83	0.74–0.92
		MC optimized performance	89.87	88.07–91.66
Anodal *n* = 16	I.	PC optimized reaction time	1.05	0.94–1.15
		PC optimized performance	91.12	88.93–93.32
		MC optimized reaction time	0.98	0.87–1.09
		MC optimized performance	91.25	89.05–93.45
	II.	PC optimized reaction time	0.97	0.88–1.06
		PC optimized performance	91.24	89.44–93.04
		MC optimized reaction time	0.89	0.80–0.98
		MC optimized performance	97.00	95.20–93.55

*Rows marked with I indicate values before, with II indicate values after stimulation*.

## Results

Before the stimulation, the three groups of volunteers performed the task under identical conditions (*n* = 48). Paired *t*-test was used for the statistical evaluation. The percentage of correct answers was 91.50 ± *SD* = 4.05 using MC stimuli, comparing with accuracy of PC stimuli (mean 90.06, ± *SD* = 4.69) the difference was not significant *p* = 0.12 (*df* = 47, *t* = 1.58, Figure 4A). Decisions about stimuli optimized for the MC yielded shorter response latencies than those for PC stimuli (mean MC latency = 0.90 s, ±*SD* = 0.20 s, mean *PC* = 0.98 s, ± *SD* = 0.23 s, *p* < 0.01, *df* = 47, *t* = −3.95, Figure 4B). These results suggest that the reaction time differences originate from the different processing times needed for MC and PC optimized stimuli, not from the differences in the recognizability of the MC and PC stimuli sets. This test verified that MC optimized stimuli are associated with shorter response latencies (Bar et al., 2006).

### Response latencies

A repeated measures three-way ANOVA was used to test main effects and possible interactions between changes in response latencies according to the types of stimulation. The within factors were the pathway (MC, PC), time of the behavioral test (before and after the stimulation) and group factor was type of stimulation (anodal, cathodal, and sham). All possible interaction terms were taken into account. Concerning the response latency times we did not find significant effects in the cases of stimulation type [*F*_(2, 45)_ = 1.336, *p* = 0.273, partial eta-squared = 0,06]. The reaction times showed differences according to the pathway factor [*F*_(1, 45)_ = 28.46, *p* < 0.01, partial eta squared = 0.39] and the time factor [*F*_(1, 45)_ = 8.69, *p* < 0.01, partial eta-squared = 0.16]. The after stimulation reaction times became faster in the case of all stimulus type, and the response latencies for MC stimuli were faster throughout the test. While analyzing the interactions, we did not find interaction between the pathway and stimulation type factor [*F*_(2, 45)_ = 0.59, *p* = 0.56, partial eta-squared = 0.03], time and stimulation type factor [*F*_(2, 45)_ = 0.36, *p* = 0.69, partial eta-squared = 0.016] and pathway and time factors [*F*_(1, 45)_ = 0.65, *p* = 0.42, partial eta-squared = 0.014]. Furthermore, there was no significant interaction between the three factors examined [*F*_(2, 45)_ = 1.99, *p* = 0.15, partial eta-squared = 0.81] (Figure 5).

### Accuracy changes

To test how transcranial stimulation of the OFC affected accuracy levels three-way ANOVA with repeated measures was used to test main effects and possible interactions between the changes in accuracy and types of stimulation. The factors again were the pathway (MC-PC), type of stimulation and time (before or after the stimulation). All possible interaction terms were taken into account. The interaction of all factors was significant [*F*_(2, 45)_ = 5.81, *p* < 0.01, partial eta-squared = 0.21]. Using stimulation type factor we found significant difference between the groups [*F*_(2, 45)_ = 4.77, *p* < 0.01, partial eta-squared = 0.18]. In the case of pathway factor we also found significant difference [*F*_(1, 45)_ = 13.74, *p* < 0.01, partial eta-squared = 0.23], but the interaction of the aforementioned factors was not significant [*F*_(2, 45)_ = 1.03, *p* = 0.36, partial eta-squared = 0.04]. Examining the effect of time factor we did not find significant differences [*F*_(1, 45)_ = 1.79, *p* = 0.19, partial eta squared = 0.04]. The interaction of time and stimulation type factor was significant [*F*_(2, 45)_ = 9.64, *p* < 0.01, partial eta-squared = 0.30] but there were no significant interactions between the time and pathway factors [*F*_(1, 45)_ = 2.78, *p* = 0.10, partial eta-squared = 0.06]. The existence of the three-factor interaction suggests that the interaction between time and stimulation depends on the level of pathway factor (PC and MC stimuli, representing two levels), with other words, the dependence between change in time and the stimulation (representing three levels) differs in the PC and MC stimuli, therefore the relationship between change in time and stimulation was evaluated at the levels of stimulus presented in the figure below. Estimated marginal means and confidence intervals in the figure are based on the results of the omnibus ANOVA (Figure 6).

We used Bonferroni *post-hoc* test to examine between which groups and conditions the significant effect can be found. The most important differences were found between accuracies measured before and after stimulation when presenting MC stimuli and using anodal (*p* < 0.01) and cathodal stimulation (*p* = 0.015). The accuracy increased when anodal stimulation was used, while the cathodal stimulation decreased the percentage of correct answers. Comparing on the level of pathway factor we found significant differences between the sham group after stimulation values (*p* < 0.01) and anodal group after stimulation values (*p* < 0.01). Furthermore, there were differences between the different groups, the accuracy for the MC stimuli after the stimulation differed between the sham and anodal groups (*p* < 0.01) and anodal and cathodal groups (*p* < 0.01). Also the accuracies measured after the stimulation using PC stimuli differed between the sham and anodal groups (*p* < 0.05).

## Discussion

Here we report that we could selectively modulate the processing of magnocellular optimized stimuli by influencing the activity of the prefrontal cortex using tDCS. This result confirms the hypothesis that magnocellular information passes the orbitofrontal cortex, and therefore might be used for a top-down modulation of visual processing.

Several points have to be addressed when discussing the results.

The first question is whether our stimuli fit for the magno- and parvocellular pathways? It has been reported earlier that decisions concerning MC optimized stimuli are faster than those optimized for PC stimuli (Kveraga et al., 2007a,b). Our results confirmed that the stimuli used in this study are indeed suitable for driving the dorsal or ventral pathway specifically. The significant difference in response latency times *before the stimulation* favored MC optimized stimuli but did not favor PC optimized stimuli, indicating that pathway optimization was successful.

TDCS had a clear and significant effect on response accuracies. How can this be interpreted? The rationale behind our study was that transcranial stimulation may have a direct impact on baseline cortical excitability (Stagg and Nitsche, 2011) and the observation that predictions might accelerate the perception of our environment by pre-stretching or priming bottom-up processing. Most studies agree that the phenomenon is based on the information carried by the MC. The MC and the dorsal pathway, however, also feed information into the ventral, PC through different stages of the cortical visual system (Merigan et al., 1993; Chen et al., 2007) but it is not clear what the exact source of this information is. Is MC information processed simultaneously, together with PC information in the ventral pathway (Mace et al., 2005; Fabre-Thorpe, 2011) or does MC information arrive through top-down connections at the IT via the OFC (Bar et al., 2006; Kveraga et al., 2007a,b)? The question is further complicated by the observation that connections between areas V5, V4 and the IT, furthermore between the prefrontal cortex and the IT can facilitate object recognition (Tomita et al., 1999; Chen et al., 2007; Eger et al., 2007). Cathodal stimulation of the OFC exerts an inhibitory effect, since neurons under the stimulation electrode become less excitable and presumably decrease the level of neurotransmitter glutamate (Filmer et al., 2014). Anodal stimulation in our experiments supported OFC functions: accuracy improved considerably for LSF stimuli (HSF stimuli were not affected), while cathodal stimulation decreased accuracy. This is in line with the meta-analysis data reported by Jacobson et al. (2012), namely, in cognitive tasks anodal stimulation often improves performance. Also, several studies report a decrease in performance when applying cathodal stimulation (e.g., Stone and Tesche, 2009; Sparing et al., 2009; Kraft et al., 2010). While this might not be the case in general, i.e., that anodal stimulation improves, cathodal stimulation impairs cognitive function, in some cognitive fields like perception and attention studies the likelihood to get opposite effects after anodal and cathodal stimulation, respectively, is exceptionally high (Jacobson et al., 2012).

The OFC consists of two large regions: medial and lateral parts. The former plays a role in higher cognitive functions, associative, reward linked learning, processing emotions, integrating sensory modalities and, most importantly, making decisions (Kringelbach and Rolls, 2004; Wallis, 2012). The fact that stimulation affected only decisions about LSF images supports the idea that magnocellular information passes the OFC. According to Bar et al. (2006) this information might be used for top-down facilitation of decision making. The role of the OFC in decision making especially when previous knowledge or predictions are concerned was studied in fMRI experiments (Summerfield et al., 2006; Miall et al., 2014; Erez and Duncan, 2015).

The last question is how tDCS influences the motor cortex and thus behavioral response latencies? Response latency in psychophysical studies includes sensory processing, decision making and motor response. When interpreting our results, one must also consider that the arrangement of electrodes for modulating the OFC (Manuel et al., 2014) stimulates the motor cortex when cathodal stimulation is used, but inhibits it when anodal stimulation is applied. Results regarding the effects of tDCS on motor reactions are far from clear. The main effect of tDCS is biasing cortical excitability. The underlying mechanism is still debated but current work suggests that it shares similarities with the activity-dependent synaptic plasticity (Dayan et al., 2013). Most studies agree that there is a large variability among subjects when evaluating the effects of stimulation (e.g., Wiethoff et al., 2014; Pope et al., 2015; Davidson et al., 2016). The situation is further complicated by the fact that the same stimulating pair of electrodes will have obviously opposing effects on the motor cortex and on the OFC; factors influencing the motor component of the decision and responding process thus might mask the effects on the sensory part. In a meta-analytical review Jacobson et al. (2012) concluded, that it is quite common to see the AeCi effect (anodal stimulation, cathodal inhibition) on latency times in motor experiments where evoked potentials are studied; in this respect our study might be an exception, since no significant differences in response latencies could be shown. We have to note however, that only behavioral response latencies and no evoked potentials were analyzed in this study.

In summary, our behavioral results show that using these electrode positions we could modulate the cortical activity of the OFC, which has an effect on the top-down mechanism during the fast categorization of MC optimized stimuli (Bar et al., 2006). Our results do not exclude the possibility that magnocellular input fed into the ventral pathway may accelerate visual processing, but they give further evidence for the essential role of top-down processes originating from the OFC in visually based decisions. The goal of our study was to investigate the effects of bilateral stimulation of the orbitofrontal cortex, but for the correct interpretation of the reaction time changes another electrode arrangement is needed. Using electrodes on the two sides of the supraorbital region (Kincses et al., 2004; Fecteau et al., 2007; Ferrari et al., 2015) could enable the examination of dynamic changes of magnocellular processing and the differences between the function of the left and right OFC. However, the exact neuronal background and tracking the flow of information along the cortical pathways require electrophysiological methods (extracellular unit recording at several locations simultaneously) with a good temporal resolution.

## Author contributions

AB: design of the work, critical revision, final approval, accountable for all aspects GCs: data acquisition, first draft, final approval, accountable for all aspects. MN: data acquisition, critical revision, approval, accountable for all aspects. PC: intrepretation of data, draft, approval, accountable for all aspects TK: intrepretation of data, draft, approval, accountable for all aspects GyS: statistical analysis, draft, approval, accountable for all aspects.

### Conflict of interest statement

The authors declare that the research was conducted in the absence of any commercial or financial relationships that could be construed as a potential conflict of interest.

## References

[B1] Antal A. Kincses T. Z. Nitsche M. A. Bartfai O. Paulus W. (2004a). Excitability changes induced in the human primary visual cortex by transcranial direct current stimulation: direct electrophysiological evidence. Invest. Ophthalmol. Vis. Sci. 45, 702–707. 10.1167/iovs.03-0688 14744917

[B2] Antal A. Kincses T. Z. Nitsche M. A. Paulus W. (2003a). Manipulation of phosphene thresholds by transcranial direct current stimulation in man. Exp. Brain. Res. 150, 375–378. 10.1007/s00221-003-1459-8 12698316

[B3] Antal A. Kincses T. Z. Nitsche M. A. Paulus W. (2003b). Modulation of moving phosphene thresholds by transcranial direct current stimulation of V1 in human. Neuropsychologia 41, 1802–1807. 10.1016/S0028-3932(03)00181-7 14527543

[B4] Antal A. Nitsche M. A. Kincses T. Z. Kruse W. Hoffmann K. P. Paulus W. (2004c). Facilitation of visuo-motor learning by transcranial direct current stimulation of the motor and extrastriate visual areas in humans. Eur. J. Neurosci. 19, 2888–2892. 10.1111/j.1460-9568.2004.03367.x 15147322

[B5] Antal A. Nitsche M. A. Kruse W. Kincses T. Z. Hoffmann K. P. Paulus W. (2004b). Direct current stimulation over V5 enhances visuo-motor coordination by improving motion perception in humans. J. Cogn. Neurosci. 16, 521–527. 10.1162/089892904323057263 15165345

[B6] Antal A. Nitsche M. A. Paulus W. (2001). External modulation of visual perception in humans. Neuroreport. 12, 3553–3555. 10.1097/00001756-200111160-00036 11733710

[B7] Antal A. Nitsche M. A. Paulus W. (2003c). Transcranial magnetic and direct current stimulation of the visual cortex. Suppl. Clin. Neurophysiol. 56, 291–304. 10.1016/S1567-424X(09)70233-8 14677406

[B8] Antal A. Paulus W. Nitsche M. A. (2011). Electrical stimulation and visual network plasticity. Restor. Neurol. Neurosci. 29, 365–374. 10.3233/RNN-2011-0609 22124032

[B9] Bar M. (2003). A cortical mechanism for triggering top-down facilitation in visual object recognition. J. Cogn. Neurosci. 15, 600–609. 10.1162/089892903321662976 12803970

[B10] Bar M. Kassam K. Ghuman A. Boshyan J. Schmid A. Dale A.. (2006). Top-down facilitation of visual recognition. Proc. Natl. Acad. Sci. U.S.A. 103, 449. 10.1073/pnas.0507062103 16407167 PMC1326160

[B11] Bar M. Tootell R. B. Schacter D. L. Greve D. N. Fischl B. Mendola J. D.. (2001). Cortical mechanisms specific to explicit visual object recognition. Neuron 29, 529–535. 10.1016/S0896-6273(01)00224-0 11239441

[B12] Bindman L. J. Lippold O. C. Redfearn J. W. (1964). The action of brief polarizing currents on the cerebral cortex of the rat (1) during current flow and (2) in the production of long-lasting after-effects. J. Physiol. 72, 369–382. 10.1113/jphysiol.1964.sp007425 PMC1368854 14199369

[B13] Brainard D. H. (1997). The pschychophysics toolbox. Vis. Res. 433–436. 9176952

[B14] Brodeur M. B. Dionne-Dostie E. Montreuil T. Lepage M. (2010). The Bank of Standardized Stimuli (BOSS), a new set of 480 normative photos of objects to be used as visual stimuli in cognitive research. PLoS ONE 5:e10773. 10.1371/journal.pone.0010773 20532245 PMC2879426

[B15] Cavada C. Company T. Tejedor J. Cruz-Rizzolo R. J. Reinoso-Suarez F. (2000). The anatomical connections of the macaque monkey orbitofrontal cortex. A review. Cereb. Cortex 10, 220–242. 10.1093/cercor/10.3.220 10731218

[B16] Cavada C. Goldman-Rakic P. S. (1989). Posterior parietal cortex in rhesus monkey: II. Evidence for segregated corticocortical networks linking sensory and limbic areas with the frontal lobe. J. Comp. Neurol. 287, 422–445. 10.1002/cne.902870403 2477406

[B17] Chen C. M. Lakatos P. Shah A. S. Mehta A. D. Givre S. J. Javitt D. C.. (2007). Functional anatomy and interaction of fast and slow visual pathways in macaque monkeys. Cereb. Cortex 17, 1561–1569. 10.1093/cercor/bhl067 16950866

[B18] Chi R. P. Fregni F. Snyder A. W. (2010). Visual memory improved by non-invasive brain stimulation. Brain Res. 1353, 168–175. 10.1016/j.brainres.2010.07.062 20682299

[B19] Costa T. L. Lapenta O. M. Boggio P. S. Ventura D. F. (2015). Transcranial direct current stimulation as a tool in the study of sensory-perceptual processing. Atten. Percept. Psychophys. 77, 1813–1840. 10.3758/s13414-015-0932-3 26139152

[B20] Costa T. L. Nagy B. V. Barboni M. T. Boggio P. S. Ventura D. F. (2012). Transcranial direct current stimulation modulates human color discrimination in a pathway-specific manner. Front. Psychiatry 3:78. 10.3389/fpsyt.2012.00078 22988446 PMC3439847

[B21] Davidson T. W. Bolic M. Tremblay F. (2016). Predicting modulation in corticomotor excitability and in transcallosal inhibition in response to anodal transcranial direct current stimulation. Front. Hum. Neurosci. 10:49. 10.3389/fnhum.2016.00049 26913001 PMC4753313

[B22] Dayan E. Censor N. Buch E. R. Sandrini M. Cohen L. G. (2013). Non-invasive brain stimulation: from physiology to network dynamics and back. Nat. Neurosci. 16, 838–844. 10.1038/nn.3422 23799477 PMC4876726

[B23] Delorme A. Richard G. Fabre-Thorpe M. (2010). Key visual features for rapid categorization of animals in natural scenes. Front. Psychol. 1:21. 10.3389/fpsyg.2010.00021 21607075 PMC3095379

[B24] De Valois K. K. Lakshminarayanan V. Nygaard R. Schlussel S. Sladky J. (1990). Discrimination of relative spatial position. Vis. Res. 30, 1649–1660. 10.1016/0042-6989(90)90150-J 2288081

[B25] Eger E. Henson R. N. Driver J. Dolan R. J. (2007). Mechanisms of top-down facilitation in perception of visual objects studied by FMRI. Cereb. Cortex 17, 2123–2133. 10.1093/cercor/bhl119 17101690 PMC2600430

[B26] Erez Y. Duncan J. (2015). Discrimination of visual categories based on behavioral relevance in widespread regions of frontoparietal cortex. J. Neurosci. 35, 12383–12393. 10.1523/JNEUROSCI.1134-15.2015 26354907 PMC4563032

[B27] Fabre-Thorpe M. (2011). The characteristics and limits of rapid visual categorization. Front. Psychol. 2:243. 10.3389/fpsyg.2011.00243 22007180 PMC3184650

[B28] Fabre-Thorpe M. Delorme A. Marlot C. Thorpe S. (2001). A limit to the speed of processing in ultra-rapid visual categorization of novel natural scenes. J. Cogn. Neurosci. 13, 171–180. 10.1162/089892901564234 11244543

[B29] Fabre-Thorpe M. Richard G. Thorpe S. J. (1998). Rapid categorization of natural images by rhesus monkeys. Neuroreport 9, 303–308. 9507973 10.1097/00001756-199801260-00023

[B30] Fang P. C. Stepniewska I. Kaas J. H. (2005). Ipsilateral cortical connections of motor, premotor, frontal eye, and posterior parietal fields in a prosimian primate, *Otolemur garnetti*. J. Comp. Neurol. 490, 305–333. 10.1002/cne.20665 16082679

[B31] Fecteau S. Pascual-Leone A. Zald D. H. Liguori P. Théoret H. Boggio P. S.. (2007). Activation of prefrontal cortex by transcranial direct current stimulation reduces appetite for risk during ambiguous decision making. J. Neurosci. 27, 6212–6218. 10.1523/JNEUROSCI.0314-07.2007 17553993 PMC6672163

[B32] Ferrari C. Lega C. Tamietto M. Nadal M. Cattaneo Z. (2015). I find you more attractive …after (prefrontal cortex) stimulation. Neuropsychologia 72, 87–93. 10.1016/j.neuropsychologia.2015.04.024 25912761

[B33] Ferrera V. P. Nealey T. A. Maunsell J. H. (1992). Mixed parvocellular and magnocellular geniculate signals in visual area V4. Nature 358, 756–761. 10.1038/358756a0 1508271

[B34] Filmer H. L. Dux P. E. Mattingley J. B. (2014). Applications of transcranial direct current stimulation for understanding brain function. Trends Neurosci. 37, 742–753. 10.1016/j.tins.2014.08.003 25189102

[B35] Goodale M. A. Milner A. D. (1992). Separate visual pathways for perception and action. Trends Neurosci. 15, 20–25. 10.1016/0166-2236(92)90344-8 1374953

[B36] Jacobson L. Koslowsky M. Lavidor M. (2012). tDCS polarity effects motor and cognitive domanis: meta-analytical review. Exp. Brain Res. 216, 1–10. 10.1007/s00221-011-2891-9 21989847

[B37] Keller F. S. Soenfeld W. N. (1950). Principles of Psychology: A Systematic Text in the Science of Behavior. New York, NY: Appleton-Century-Crofts.

[B38] Kincses T. Z. Antal A. Nitsche M. A. Bartfai O. Paulus W. (2004). Facilitation of probabilistic classification learning by transcranial direct current stimulation of the prefrontal cortex in the human. Neuropsychologia 42, 113–117. 10.1016/S0028-3932(03)00124-6 14615081

[B39] Knierim J. J. van Essen D. C. (1992). Neuronal responses to static texture patterns in area V1 of the alert macaque monkey. J. Neurophysiol. 67, 961–980. 1588394 10.1152/jn.1992.67.4.961

[B40] Kraft A. Roehmel J. Olma M. C. Schmidt S. Irlbacher K. Brandt S. A. (2010). Transcranial direct current stimulation affects visual perception measured by threshold perimetry. Exp. Brain Res. 207, 283–290. 10.1007/s00221-010-2453-6 21046369

[B41] Kringelbach M. L. Rolls E. T. (2004). The functional neuroanatomy of the human orbitofrontal cortex: evidence from neuroimaging and neuropsychology. Prog. Neurobiol. 72, 341–372. 10.1016/j.pneurobio.2004.03.006 15157726

[B42] Kveraga K. Boshyan J. Bar M. (2007a). Magnocellular projections as the trigger of top-down facilitation in recognition. J. Neurosci. 27, 13232–13240. 10.1523/JNEUROSCI.3481-07.2007 18045917 PMC6673387

[B43] Kveraga K. Ghuman A. S. Bar M. (2007b). Top-down predictions in the cognitive brain. Brain Cogn. 65, 145–168. 10.1016/j.bandc.2007.06.007 17923222 PMC2099308

[B44] Lin F. H. Witzel T. Hamalainen M. S. Dale A. M. Belliveau J. W. Stufflebeam S. M. (2004). Spectral spatiotemporal imaging of cortical oscillations and interactions in the human brain. Neuroimage 23, 582–595. 10.1016/j.neuroimage.2004.04.027 15488408 PMC2884198

[B45] Mace M. J. Delorme A. Richard G. Fabre-Thorpe M. (2010). Spotting animals in natural scenes: efficiency of humans and monkeys at very low contrasts. Anim. Cogn. 13, 405–418. 10.1007/s10071-009-0290-4 19921288

[B46] Mace M. J. Thorpe S. J. Fabre-Thorpe M. (2005). Rapid categorization of achromatic natural scenes: how robust at very low contrasts? Eur. J. Neurosci. 21, 2007–2018. 10.1111/j.1460-9568.2005.04029.x 15869494

[B47] Manuel A. L. David A. W. Bikson M. Schnider A. (2014). Frontal tDCS modulates orbitofrontal reality filtering. Neuroscience 265, 21–27. 10.1016/j.neuroscience.2014.01.052 24508152

[B48] Maunsell J. H. Newsome W. T. (1987). Visual processing in monkey extrastriate cortex. Annu Rev. Neurosci. 10, 363–401. 10.1146/annurev.ne.10.030187.002051 3105414

[B49] Merigan W. H. Nealey T. A. Maunsell J. H. (1993). Visual effects of lesions of cortical area V2 in macaques. J. Neurosci. 13, 3180–3191. 8331392 10.1523/JNEUROSCI.13-07-03180.1993 PMC6576679

[B50] Miall R. C. Nam S. H. Tchalenko J. (2014). The influence of stimulus format on drawing a functional imaging study of decision making in portrait drawing. Neuroimage 2, 608–619. 10.1016/j.neuroimage.2014.08.015 PMC4229501 25128710

[B51] Mishkin M. Ungerleider L. G. (1982). Contribution of striate inputs to the visuospatial functions of parieto-preoccipital cortex in monkeys. Behav. Brain Res. 6, 57–77. 10.1016/0166-4328(82)90081-X 7126325

[B52] Nealey T. A. Maunsell J. H. (1994). Magnocellular and parvocellular contributions to the responses of neurons in macaque striate cortex. J. Neurosci. 14, 2069–2079. 8158257 10.1523/JNEUROSCI.14-04-02069.1994 PMC6577134

[B53] Nitsche M. A. Cohen L. G. Wassermann E. M. Priori A. Lang N. Antal A.. (2008). Transcranial direct current stimulation: state of the art 2008. Brain Stimul. 1, 206–223. 10.1016/j.brs.2008.06.004 20633386

[B54] Nitsche M. A. Paulus W. (2000). Excitability changes induced in the human motor cortex by weak transcranial direct current stimulation. J. Physiol. 3, 633–639. 10.1111/j.1469-7793.2000.t01-1-00633.x PMC2270099 10990547

[B55] Nitsche M. A. Paulus W. (2001). Sustained excitability elevations induced by transcranial DC motor cortex stimulation in humans. Neurology 57, 1899–1901. 10.1212/WNL.57.10.1899 11723286

[B56] Nowak L. G. Bullier J. (1997). The timing of information transfer in the visual system, in Extrastriate Cortex in Primates, eds Rockland K. S. Kaas J. H. Peters A. (New York, NY: Springer), 205–241.

[B57] Nowak L. G. Munk M. H. Girard P. Bullier J. (1995). Visual latencies in areas V1 and V2 of the macaque monkey. Vis. Neurosci. 12, 371–384. 10.1017/S095252380000804X 7786857

[B58] Oldfield R. C. (1971). The assessment and analysis of handedness: the Edinburgh inventory. Neuropsychologia 9, 97–113. 10.1016/0028-3932(71)90067-4 5146491

[B59] Pope P. A. Brenton J. W. Miall R. C. (2015). Task-specific facilitation of cognition by anodal transcranial direct current stimulation of the prefrontal cortex. Cereb. Cortex 11, 4551–4558. 10.1093/cercor/bhv094 PMC4816797 25979089

[B60] Purpura D. P. McMurtry J. G. (1965). Intracellular activities and evoked potential changes during polarization of motor cortex. J. Neurophysiol. 28, 166–185. 14244793 10.1152/jn.1965.28.1.166

[B61] Rokszin A. A. Győri-Dani D. Nyúl L. G. Csifcsák G. (2016). Electrophysiological correlates of top-down effects facilitating natural image categorization are disrupted by the attenuation of low spatial frequency information. Int. J. Psychophysiol. 100, 19–27. 10.1016/j.ijpsycho.2015.12.006 26707649

[B62] Sachs M. B. Nachmias J. Robson J. G. (1971). Spatial-frequency channels in human vision. J. Opt. Soc. Am. 61, 1176–1186. 10.1364/JOSA.61.001176 5121888

[B63] Schmolesky M. T. Wang Y. Hanes D. P. Thompson K. G. Leutgeb S. Schall J. D.. (1998). Signal timing across the macaque visual system. J. Neurophysiol. 79, 3272–3278. 9636126 10.1152/jn.1998.79.6.3272

[B64] Schyns P. G. Oliva A. (1994). From blobs to boundary edges: evidence for time- and spatial-scale-dependent scene recognition. Psychol. Sci. 195–200. 10.1111/j.1467-9280.1994.tb00500.x

[B65] Sparing R. Thimm M. Hesse M. D. Küst J. Karbe H. Fink G. R. (2009). Bidirectional alterations of interhemispheric parietal balance by non-invasive cortical stimulation. Brain 132, 3011–3020. 10.1093/brain/awp154 19528092

[B66] Stagg C. J. Nitsche M. A. (2011). Physiological basis of transcranial direct current stimulation. Neuroscientist 1, 37–53. 10.1177/1073858410386614 21343407

[B67] Stone D. B. Tesche C. D. (2009). Transcranial direct current stimulation modulates shifts in global/local attention. Neuroreport 20, 1115–1119. 10.1097/WNR.0b013e32832e9aa2 19590395

[B68] Sugase Y. Yamane S. Ueno S. Kawano K. (1999). Global and fine information coded by single neurons in the temporal visual cortex. Nature 400, 869–873. 10.1038/23703 10476965

[B69] Summerfield C. Egner T. Greene M. Koechlin E. Mangels J. Hirsch J. (2006). Predictive codes for forthcoming perception in the frontal cortex. Science 5803, 1311–1314. 10.1126/science.1132028 17124325

[B70] Tamura H. Tanaka K. (2001). Visual response properties of cells in the ventral and dorsal parts of the macaque inferotemporal cortex. Cereb. Cortex 11, 384–399. 10.1093/cercor/11.5.384 11313291

[B71] Thorpe S. J. Fabre-Thorpe M. (2003). Fast visual processing and its implications, in The Handbook of Brain Theory and Neural Networks, ed Arbib M. A. (Cambridge: MIT press), 441–444.

[B72] Thorpe S. J. Gegenfurtner K. R. Fabre-Thorpe M. Bulthoff H. H. (2001). Detection of animals in natural images using far peripheral vision. Eur. J. Neurosci. 14, 869–876. 10.1046/j.0953-816x.2001.01717.x 11576191

[B73] Tomita H. Ohbayashi M. Nakahara K. Hasegawa I. Miyashita Y. (1999). Top-down signal from prefrontal cortex in executive control of memory retrieval. Nature 401, 699–703. 10.1038/44372 10537108

[B74] Tootell R. B. Hamilton S. L. Switkes E. (1988). Functional anatomy of macaque striate cortex. IV. Contrast and magno-parvo streams. *J*. Neurosci. 8, 1594–1609. 10.1523/JNEUROSCI.08-05-01594.1988 PMC6569196 3367212

[B75] Varga E. T. Elif K. Antal A. Zimmer M. Harza I. Paulus W.. (2007). Cathodal transcranial direct current stimulation over the parietal cortex modifies facial gender adaptation. Ideggyogy Sz. 60, 474–479. 18198794

[B76] Wallis J. D. (2012). Cross-species studies of orbitofrontal cortex and value-based decision-making. Nat. Neurosci. 15, 13–19. 10.1038/nn.2956 22101646 PMC3549638

[B77] Wiethoff S. Hamada M. Rothwell J. C. (2014). Variability in response to transcranial direct current stimulation of the motor cortex. Brain Stimul. 3, 468–475. 10.1016/j.brs.2014.02.003 24630848

[B78] Willenbockel V. Sadr J. Fiset D. Horne G. O. Gosselin F. Tanaka J. W. (2010). Controlling low-level image properties: the SHINE toolbox. Behav. Res. Methods 42, 671–684. 10.3758/BRM.42.3.671 20805589

[B79] Willis M. L. Murphy J. M. Ridley N. J. Vercammen A. (2015). Anodal tDCS targeting the right orbitofrontal cortex enhances facial expression recognition. Soc. Cogn. Affect. Neurosci. 10, 1677–1683. 10.1093/scan/nsv057 25971602 PMC4666107

[B80] Zipser K. Lamme V. A. Schiller P. H. (1996). Contextual modulation in primary visual cortex. J. Neurosci. 16, 7376–7389. 8929444 10.1523/JNEUROSCI.16-22-07376.1996 PMC6578953

